# Hysteroembryoscopy in the management of recurrent early pregnancy loss

**DOI:** 10.1007/s00404-025-08018-2

**Published:** 2025-04-15

**Authors:** Ismet Hortu, Cihan Mutu

**Affiliations:** Department of Obstetrics and Gynecology, School of Medicine, Ege University, 35100 Izmir, Turkey

**Keywords:** Hysteroscopy, Embryoscopy, Hysteroembryoscopy

## Introduction

Spontaneous miscarriage during the first trimester occurs in approximately 15% of all pregnancies [1]. The evacuation of pregnancy products using hysteroscopy in cases of early pregnancy loss has been shown to be a safe and effective technique. In selected cases, hysteroembryoscopy emerges as an innovative alternative surgical option for managing early pregnancy loss [2].

## Case presentation

This article aims to illustrate the steps involved in hysteroembryoscopic evaluation through the case of a 28-year-old woman (G4P1A2) with a negative thrombophilia panel. She has a history of two missed abortions and recently experienced a pregnancy loss at 8 weeks. Her previous pregnancy losses occurred at 8 and 12 weeks, both of which required suction curettage. Video 1 demonstrates the procedure performed.

**Fig. 1:**
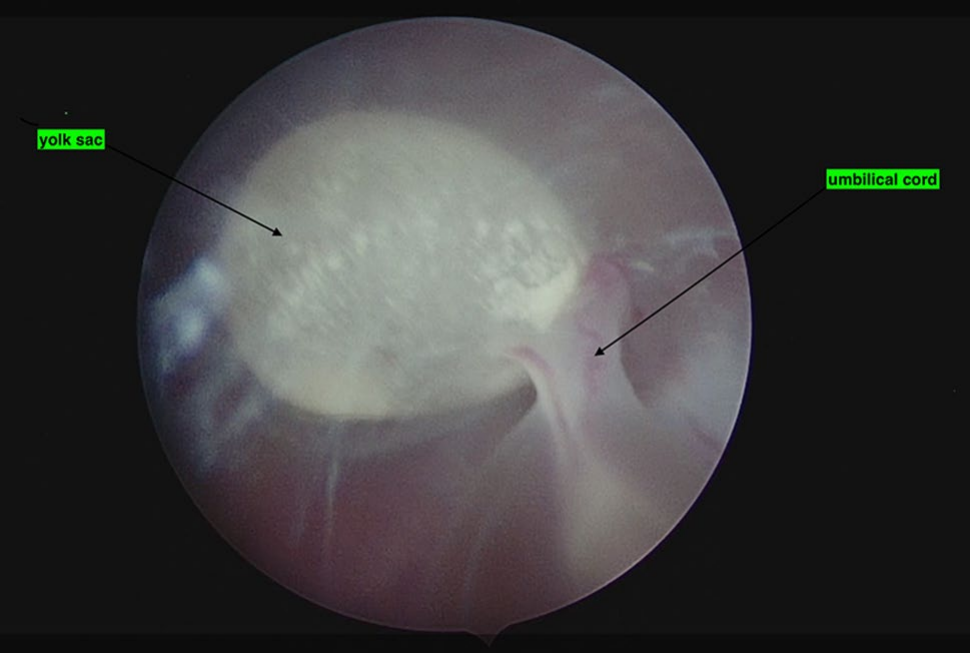
The yolk sac and umbilical cord

**Fig. 2:**
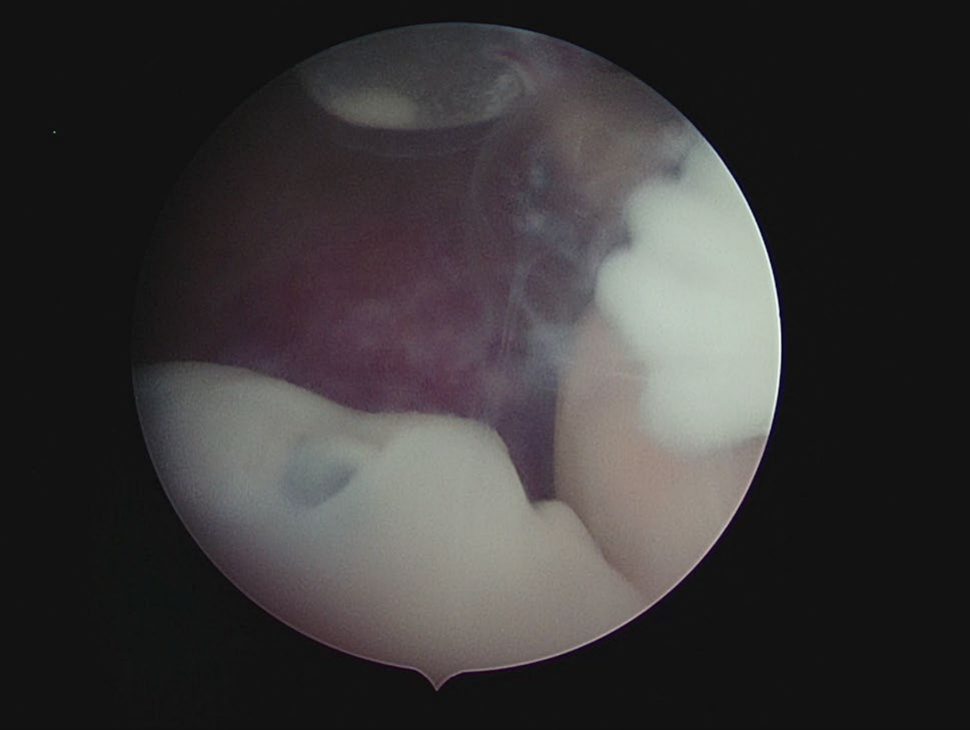
The embryo’s face and the embryo’s right hand

In this patient, who had no significant comorbidities and a history of two early pregnancy losses, hysteroembryoscopy was presented as a viable treatment option. This approach aimed to facilitate karyotype analysis and minimize the risk of retained products of conception. Figure 1 illustrates the yolk sac and umbilical cord of the missed fetus, while Fig. 2 shows the face of the embryo.

## Discussion

Recurrent miscarriages are associated with various obstetric complications in future pregnancies and can impose significant financial and psychosocial burdens on affected individuals. Therefore, advancements in treatment options are essential in this field. The endoscopic approach of hysteroembryoscopy offers several benefits, including lower maternal cell contamination compared to traditional blind curettage, more complete tissue removal, reduce risk of uterine perforation and reduced intrauterine adhesion formation [3, 4].

When evaluating the cost-effectiveness of hysteroembryoscopy compared to classic curettage, using equipments for hysteroembryoscopy is more expensive than classic curettage, but hysteroembryoscopy is more effective for evaluation of recurrent early pregnancy loss. Management of recurrent early pregnancy loss hysteroscopic evaluation of the uterine cavity is frequently requires, and hysteroembryoscopy is provide us evaluation of the uterine cavity. Fayek et al. found a 24.9% prevalence of uterine anomalies in patients with recurrent pregnancy loss in a retrospective cohort study [5]. Additionally, during the hysteroembryoscopy we visualise of the bilateral tubal ostiums. For all these reasons in management of recurrent early pregnancy loss the cost effectiveness of hysteroembryoscopy is more efficient than classic curettage.

The first difference between hysteroscopy and hysteroembryoscopy is pressure of the optical medium fluid. In hysteroembryoscopy, this pressure is lower, typically around 60 mmH2O, compared to 100 mmH20 in hysteroscopy. The second difference is hysteroscopy requires a 10 mm optical hysteroscope for operative procedures such as myomectomy, and 5 mm optics are sufficient for hysteroembryoscopy. The hysteroembryoscopy focuses on morphological assessment of the embryo in contrast to hysteroscopy which mainly focuses on uterine cavity.

## Conclusion

In summary, hysteroembryoscopy represents a promising technique for evaluating and managing early pregnancy loss, particularly in patients with a history of recurrent miscarriages, and this technique is feasible, safe and inexpensive [6]. Further research and clinical application of this method could enhance outcomes for women facing similar challenges.

## Supplementary Information

Below is the link to the electronic supplementary material.

**Supplementary file 1** (MP4 22621 KB)

## Data Availability

No datasets were generated or analysed during the current study.
